# Health Promotion in Popular Web-Based Community Games Among Young People: Proposals, Recommendations, and Applications

**DOI:** 10.2196/39465

**Published:** 2023-06-09

**Authors:** Philippe Martin, Boris Chapoton, Aurélie Bourmaud, Agnès Dumas, Joëlle Kivits, Clara Eyraud, Capucine Dubois, Corinne Alberti, Enora Le Roux

**Affiliations:** 1 Université Paris Cité, Epidémiologie Clinique Evaluation Economique Appliquées aux Populations Vulnérables, Inserm Paris France; 2 Institut National d’Etudes Démographiques, UR14 – Sexual and Reproductive Health and Rights Aubervilliers France; 3 Assistance Publique des Hôpitaux de Paris, Hôpital Universitaire Robert Debré, Unité d’épidémiologie clinique, Inserm, Centre d'Investigation Clinique 1426 Paris France; 4 GDID Santé Paris France; 5 Université Jean Monnet Coactis UR 4161 Saint-Etienne France; 6 Groupe de Recherche en Médecine et Santé de l'Adolescent Paris France; 7 Cité du Genre Paris France; 8 Réseau Francophone de Littératie en Santé Paris France; 9 Fil Santé Jeunes Paris France

**Keywords:** health promotion, web-based community games, young people, interventional research, recommendations

## Abstract

**Background:**

Young people use digital technology on a daily basis and enjoy web-based games that promote social interactions among peers. These interactions in web-based communities can develop social knowledge and life skills. Intervening via existing web-based community games represents an innovative opportunity for health promotion interventions.

**Objective:**

The aim of this study was to collect and describe players’ proposals for delivering health promotion through existing web-based community games among young people, elaborate on related recommendations adapted from a concrete experience of intervention research, and describe the application of these recommendations in new interventions.

**Methods:**

We implemented a health promotion and prevention intervention via a web-based community game (Habbo; Sulake Oy). During the implementation of the intervention, we conducted an observational qualitative study on young people’s proposals via an intercept web-based focus group. We asked 22 young participants (3 groups in total) for their proposals about the best ways to carry out a health intervention in this context. First, using verbatim transcriptions of the players’ proposals, we conducted a qualitative thematic analysis. Second, we elaborated on recommendations for action development and implementation based on our experiences and work with a multidisciplinary consortium of experts. Third, we applied these recommendations in new interventions and described their application.

**Results:**

A thematic analysis of the participants’ proposals revealed 3 main themes and 14 subthemes related to their proposals and process elements: the conditions for developing an attractive intervention within a game, the value of involving peers in developing the intervention, and the ways to mobilize and monitor gamers’ participation. These proposals emphasized the importance of interventions involving and moderating a small group of players in a playful manner but with professional aspects. We established 16 domains with 27 recommendations for preparing an intervention and implementing it in web-based games by adopting the codes of game culture. The application of the recommendations showed their usefulness and that it was possible to make adapted and diverse interventions in the game.

**Conclusions:**

Integrated health promotion interventions in existing web-based community games have the potential for promoting the health and well-being of young people. There is a need to incorporate specific key aspects of the games and gaming community recommendations, from conception to implementation, to maximize the relevance, acceptability, and feasibility of the interventions integrated in current digital practices.

**Trial Registration:**

ClinicalTrials.gov NCT04888208; https://clinicaltrials.gov/ct2/show/NCT04888208

## Introduction

### Background

Health promotion requires the creation of health-enhancing public policy with diverse and complementary approaches, particularly in the face of public health challenges (chronic diseases, social inequalities, and health systems issues) [1]. In this sense, health promotion is defined as the process of enabling people to increase their control over and improve their own health [2]. Areas for action include creating supportive environments, strengthening community actions, and developing personal skills. Health promotion interventions must then consider the importance of community and social dimensions, mobilizing social interactions as a lever for population health [3]. These social dimensions can reduce risk factors and increase the well-being of individuals.

Youth is a major period of empowerment and life evolution, with the formation of identity, social relationships, new responsibilities, and a professional future. It is also a period marked by health issues [4] that health promotion can address [2]. To do so, public health actors must understand the population’s concerns, habits, and culture to adapt their intervention strategies. Describing and understanding the characteristics of an identified group and studying its behaviors, expectations, and preferences enables the development of health actions with greater impact on the target audience and society [5,6].

Web-based games are attractive and widely used by young people, involving an environment in which players can explore, interact, and follow scenarios in association with characters and stories [7]. Games present relevant challenges for players who require different types of effort to solve tasks (ludic activities) or share experiences [7,8]. The “community” aspect of some web-based games means that, as in social network sites, players can connect with others, build web-based relationships, and find a “peer-to-peer” environment and support around common interests and shared game codes [9]. The community aspect can be centered on specific ages, lifestyles, backgrounds, or thematic characteristics such as interests, hobbies, pastimes, or games [9,10].

Some game-based health promotion interventions are promising, with positive results in terms of feasibility and short-term effects on knowledge and attitudes related to diet, physical activity, and lifestyle [7,11]. However, there is a need to address some key challenges in terms of the process and optimal conditions of implementation to understand how games can be the site of effective health promotion (intervention mechanisms) and research. First, researchers must describe the implementation means of such interventions. Poor conceptualization or bad implementation of an intervention can have a direct impact on its effectiveness [12]. To be effective, web-based games created for health promotion should include the following characteristics or features: feasibility, ease of use, storytelling, feedback, fun, and a proven focus on the health topic addressed [7]. In the case of web-based games that were not originally dedicated to health promotion and research, such data are missing.

The Scott project aimed to deliver a health promotion and disease prevention intervention via an existing web-based community game for young people. Indeed, our hypothesis was that intervening via popular games might enhance the acceptability (user-friendliness) and scope of our actions. The Scott intervention consisted of facilitating small-group health discussions in the game. We chose the Habbo game (developed by the video game company Sulake Oy)—a free, popular, and web-based game with social interactions in which it was possible to intervene.

### Goal of This Study

On the basis of the Scott concrete experience, the aim of this study was to (1) analyze players’ proposals during the implementation of the intervention in the Habbo game, (2) elaborate on researchers’ recommendations for conducting a health promotion intervention in existing web-based community games among young people, and (3) describe the application of these recommendations in new interventions in the same Habbo game.

## Methods

### Overview

Our study is embedded in the Scott project, which is broadly a health promotion intervention research project aimed at implementing and evaluating an intervention in a web-based game. The first pilot study was conducted in 2020 [13]. The Scott project is registered on ClinicalTrials.gov (identifier NCT04888208).

During the implementation phase of the intervention, we conducted this qualitative study on the expectations and proposals of the participants in the first pilot study. Of all the pilot study participants, a panel of 22 participants were present to participate in the study.

**Figure 1:**
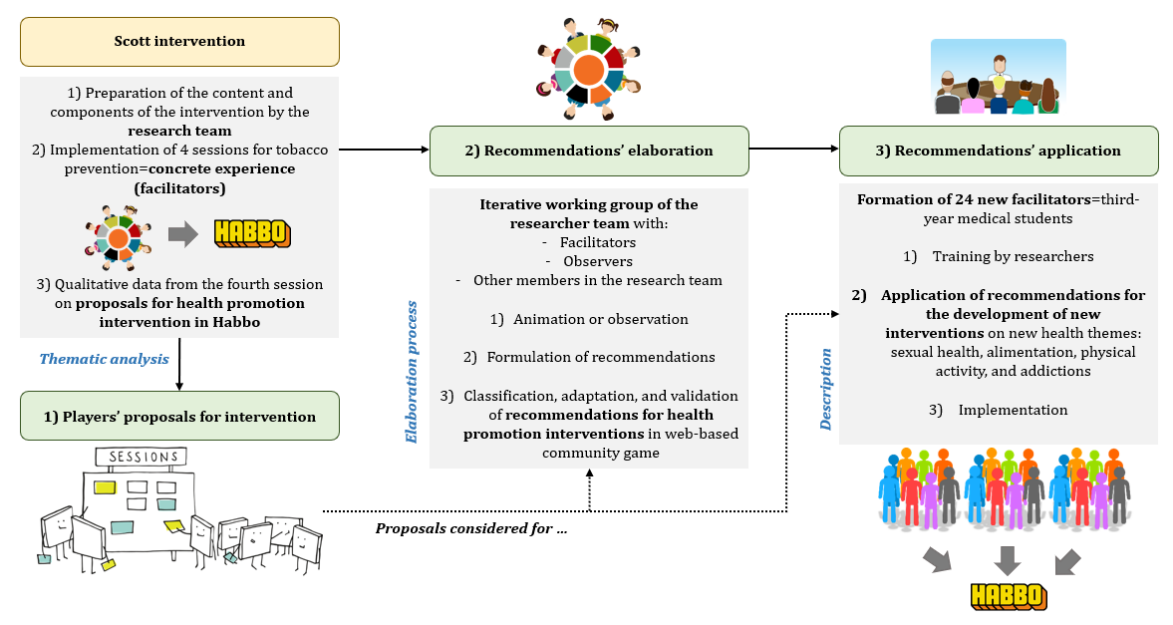
Stages and study process of the study.

We conducted a qualitative study nested in the Scott intervention implementation based on (1) a questioning of players’ proposals for delivering health promotion within a web-based community game, (2) working groups with a multidisciplinary consortium of researchers for the elaboration of recommendations (based on our concrete experience), and (3) an application of these recommendations for new interventions in the same game. The stages and study processes are shown in Figure 1.

### Scott Intervention

The Scott intervention was a health promotion and prevention intervention, initially a tobacco prevention initiative, integrated into a popular web-based community game and thus targeting adolescent and young adult game players (the game is officially permitted to be played by an audience aged ≥14 years). During this experiment, we investigated players’ proposals for pursuing health promotion within the game on any health themes.

#### Theory

The entire theoretical framework of the intervention is described in Multimedia Appendix 1 [14-20]. The theoretical framework is based on the notions of social support, group motivation, and social learning [21], as applied to small web-based groups [14]. The mobilized theories are the theory of planned behavior [15], social learning theory [16], peer education [17] (with a link between peer education and social learning theory [18]), and the notion of small-group behavior change in web-based communities [14]. The main premise is that group discussion fosters the development of collective motivation and sufficient knowledge, norms, and skills to promote favorable health behaviors.

#### Setting

We conducted the intervention in the French-speaking version of the web-based world Habbo. Habbo is an open-world web-based social network–type game with gamification elements. Habbo is a fun game with various activities with social interactions and different worlds in the game, allowing players to participate in discussion groups. The players can communicate with each other by sending limited character messages. The instant messages are limited to 50 characters. This results in many messages, a fast flow of messages, and multiple conversations in 1 space.

**Figure 2:**
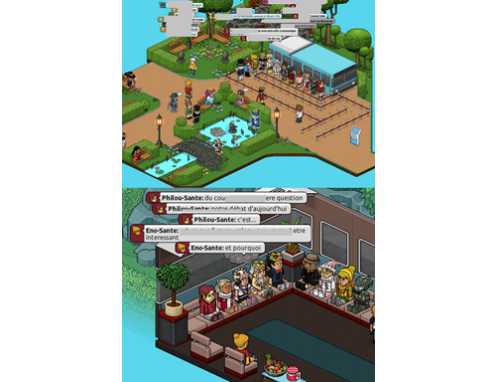
Garden and bus for the project (place of intervention).

Ambassador players are players who are recognized for their attendance and good behavior. A team of moderators and a community manager ensure that the exchanges are fair. The developers of the Habbo game already allow external health actors to intervene in their game for health promotion interventions (not involving research) with the facilitation of the community manager and ambassador players. In the Scott project, we asked for the creation of a space in the web-based game (named a “room” inside the game) specifically for the project, with the appearance of a garden inside which a bus with a maximum capacity of 10 players (small group) was parked for the delivery of the intervention (Figure 2). A queue was set up to allow a limited number of participants to get access to the bus (10 players per session). We replicated the intervention framework of Fil Santé Jeunes (a French information service for young people [aged 12-25 years] in the field of health), which has been intervening in Habbo for several years. One of the research team members (CD) facilitated contact with Sulake and the transmission of needed experiences and skills. The relationship with Sulake was established entirely through the Habbo game’s community manager. She helped us prepare the intervention (practical arrangements, recruitment, the dissemination of information notes, the implementation of the intervention, and the distribution of measurement tools). Moreover, she acted as a unique intermediary between the developer and researcher, especially with regard to the following: the temporality of the intervention, the content of the sessions, questionnaires, data collected, and feedback.

#### Facilitators

A pair of facilitators (PM and ELR) were in charge of the animation and moderation of action. This pair was composed of 2 public health researchers, identified in the game as woman and man, to ensure a gender mix. They had a pseudonym integrating the term “health” to assert their professional position.

#### Development and Preparation

We developed the intervention content with a pluridisciplinary research team consisting of prevention workers, a psychologist (already leading a support group centered on adolescents’ issues in the Habbo game), the game’s community manager, sociology and social marketing professionals, and researchers involved in health promotion and intervention research. Two researchers (the facilitators) were previously trained by one of the regular health professionals operating in the game, the community manager of the game, and 3 experienced players to facilitate the intervention. They were trained to test the game’s commands (speak, exclude, silence, etc) before setting up the intervention.

#### Involvement of Peer Players

We worked with the ambassador players for the preparation of the first intervention (part 1) and application of the recommendations (part 3). These players were involved in the training of the facilitators (getting to grips with the features) and in the construction of the places of intervention in the game (Habbo players can create different spaces on the web).

In the game, players can collect badges (with no monetary value), which were used as incentives for this project. Badges were allocated to the participants before and after participation. To enhance novelty, we proposed the opportunity to create and provide original badges dedicated to this project (coconstruction vision) to some volunteer ambassadors.

#### Contents and Processes

We offered four 30-minute discussion sessions on 4 Thursdays in October 2020 at the proposed times of 11 AM, 3 PM, and 5 PM (12 sessions). We proposed different types of content concerning health issues and tobacco prevention (session 1: representations of tobacco, session 2: benefits of not smoking, session 3: resisting proposals from those around you, and session 4: helping others by making proposals for others’ intervention). For the fourth and final session, we asked all 22 players (1 session per group; 3 groups=3 sessions) for their proposals and recommendations for pursuing health promotion actions in the game.

### Participants

The participants were all volunteer French-speaking Habbo players. A preintervention survey was conducted in January 2020 to ensure that the average age of the given sample corresponded to that of a population of adolescents and young adults. Of the 83 responses, 75% (n=62) were from male participants, and the median age of the participants was 23 years, with a minimum age of 16 years and a maximum age of 32 years. The median age is in accordance with the World Health Organization’s definition of young people’s age range, which is 10 to 24 years [4].

### Recruitment Process

We recruited study participants in October 2020. A week before the first session, we asked for a banner advertisement on the game’s log-in page with all the information about the study (objective, facilitator’s contacts, time, place, and subject). We also explained that not all players would be able to participate because of limited places and randomization. The participants were informed that they were participating in public health research. After inclusion, before the session integrating the qualitative study (session 4), the participants were informed that the purpose of this session was to collect their proposals for future interventions.

On the day of the first intervention, on the home page of the game, the participants had to fill in a questionnaire on baseline information (its completion constituted a criterion of eligibility for randomization). After the completion of the questionnaire, the participants were randomly provided either the intervention group badge or the control group badge. We planned 3 time slots for recruitment on the same day, at 11 AM, 3 PM, and 5 PM. For each time slot, a maximum of 10 participants received the intervention badge, which was valid for the 4 sessions (the day of randomization and the following 3 Thursdays). The control group, as well as the intervention group, had access to health information on preexisting websites posted in the project room.

### Data Collected

Data were collected (1) from the qualitative study and the fourth session (focus group) on proposals, (2) from the working groups with a multidisciplinary consortium of researchers and feedback from the research facilitators, and (3) from observations of other interventions developed on the basis of the recommendations and applied by other facilitators.

#### 1. Players’ Proposals for Intervention

During the last (fourth) session, the players from the intervention group were asked about the attractiveness of the intervention and their proposals for health promotion in the game. We asked them to insert health promotion and prevention interventions into the Habbo game but beyond the Scott intervention. We asked the following questions: “Would you be willing to help us do prevention activities in Habbo? What would you consider important? What would you want to implement?” We collected verbatim testimonies from messages that illustrated players’ proposals, elements of attractiveness, and the process of the intervention. No personal or identifying data (including pseudonyms) were collected for analysis. We collected the number of participants and number of messages sent during each session. Beyond these data, the facilitators collected personal feelings about the sessions, with field notes and points for animating and moderating interventions. We transcribed the participants’ testimonies and associated them with a participant code (S1, S2, ..., Si) to preserve anonymity.

#### 2. Recommendations’ Elaboration

Iterative working group sessions were held among researchers (including facilitators) to share and analyze feedback from the intervention sessions and formulate recommendations for further interventions in the games. These data were in the form of raw notes and feedback reports.

#### 3. Recommendations’ Application

Three authors (PM, ELR, and CE) applied these recommendations during the training of new intervention facilitators in the same game. PM, ELR, and CE were responsible for training and supervising these facilitators. The facilitators were (third-year) medical students carrying out a practical initiation into prevention as part of their training. Eight different interventions over 2 weeks (4 per week), with 3 facilitators per intervention (24 facilitators [third-year medical students]) and new health themes (sexual health, addiction, physical activity, food, and nutrition), were developed. They conceptualized these new interventions based on the recommendations on diverse health themes, such as addiction, nutrition, sexual health, and physical activity.

### Analysis

We conducted a qualitative analysis based on (1) the chat transcripts of the players, (2) a description of the recommendations elaborated by the researchers’ working group, and (3) a description of the observation of the recommendations’ application.

#### 1. Qualitative Analysis of “Players’ Proposals for Intervention”

We conducted a qualitative analysis of the participants’ proposals regarding elements related to health promotion interventions: context, audience expectations, intervention components, and implementation modalities [22].

We conducted a thematic analysis focusing on the proposals and process elements related to the acceptability, attractiveness, and implementation of interventions in a web-based community game specifically. We allowed the participants’ proposals to emerge during the sessions. The analysis was conducted based on raw proposals without predefined themes. The authors formulated proposals around the main themes and associated subthemes following these stages: initialization, construction, rectification, and finalization [23]. The initialization phase corresponded to the rereading of each session (thanks to the game’s conversation history). The researchers EL and PM took notes on the emerging themes and their impressions. These 2 researchers then carried out the construction phase by classifying and describing the different expectations and proposals of the participants through a first classification and organization of the themes. For the analysis, we accumulated several messages from the same participant to relate all their proposals (only if they made several short messages in a row). The rectification phase corresponded to the distancing of the themes, notably by proposing them to the other researchers of the team, for adaptation and stabilization. This phase also allowed us to put forward the themes that brought real new scientific knowledge. Finally, the finalization phase focused on organizing the themes according to the link or distance between them (organization of the “storyline” of the proposals).

#### 2. Recommendations’ Elaboration

Following the intervention, the researchers provided feedback and developed recommendations for interventions specifically conducted in web-based community games. These recommendations were designed based on current recommendations for interventional research, on the experiences of the 2 researchers involved as facilitators (PM and ELR), and on the data from researchers who acted as observers during some sessions (BC, JK, and AD); discussed; adapted; classified; and validated by all the researchers associated with the project.

We analyzed domains and recommendations: the domains correspond to the important steps of preparation, conceptualization of the intervention, and realization of the intervention (implementation); the recommendations are concrete and detailed suggestions for how to meet the expectations for each domain.

#### 3. Recommendations’ Application

We observed and formulated a description of the recommendation-based interventions developed, centered on components and implementation modalities, to illustrate the concrete application of each recommendation.

### Ethics Approval

The Inserm Ethics Review Committee and Inserm Institutional Review Board (IRB00003888, IORG0003254, and FWA00005831) provided a favorable opinion for our study and approved its information circuit on July 7, 2020.

## Results

### Overview

A total of 22 players participated in the session for the collection of proposals. Proposals were collected from groups of players (3 groups of 9, 8, and 5 players, named S1 to S22 in the following results), and 1715 messages were posted (an average of 572 messages per session), of which 1155 (67%) were posted by the participants and 560 (33%) were posted by the facilitators and researchers (PM and ELR). The facilitators and researchers (PM and ELR) who conducted the focus groups considered that the third group interviewed, in addition to the other 2 groups, provided no new information. Thus, they considered that data saturation had been reached.

### Players’ Proposals for Intervention

Thematic analysis of the participants’ proposals revealed 3 main themes and 14 subthemes related to their proposals and process elements for health promotion action within the web-based community game. Textbox 1 presents these themes.

**Key themes and subthemes identified in the session.**

**Develop an attractive intervention in the game**

- The need for varied and renewed health topics
- Entertainment components and formats for prevention
- Challenge as a motivation to participate
- An entertaining intervention that may be considered not serious enough
- The involvement of multiple stakeholders in the development and implementation of interventions

**Involve peers in the development of the intervention in the game**

- In favor of codeveloping the action
- Remaining anonymous for the fear of not being taken seriously
- Feeling of insufficient knowledge for delivering prevention themselves
- Experience as a skill for prevention
- Motivation and social support among peers as a lever for action

**Mobilize and control gamer participation**

- Limiting the number of participants to ensure the success of the action
- Trolls and essential moderation of exchanges
- In-game rewards as incentives
- Promote action outside the web-based game

#### Develop an Attractive Intervention in the Game

All the interviewed participants expressed an interest in contributing to the development of a prevention intervention in a web-based game. Through 5 subthemes, the analysis highlighted the themes, expected components, and formats, as well as the different stakeholders involved in the design and implementation of the intervention.

##### The Need for Varied and Renewed Health Topics

Initially, the participants mentioned the importance of addressing a variety of health topics. One of the participants (S2) suggested using a polling system to guide the topics addressed. Two participants mentioned the importance of including topics around “anything harmful” (S11) and health consequences by highlighting “impact features” (S2). One of the participants suggested a period for renewing topics, with “new themes each week” (S11).

##### Entertainment Components and Formats for Prevention

The participants proposed several action components, mostly participatory and interactive components, such as quizzes, games, and discussion groups (S11 and S12), to structure interventions. One of the participants (S11) explained the need to create places in the web-based game dedicated to prevention in the game, such as theaters or apartments. Another participant (S12) highlighted the importance of using animation and games as levers of participation:

There need to be animations and games. Those make it more engaging, and questionnaires are attractive too. You know, you can do everything and anything like that, with quizzes in the form of questionnaires.

S12

One of the participants mentioned the possibility of asking questions through a question box system (S11 and S12). Another suggested a system in which participants can ask questions through forums (S6). The same participant (S6) mentioned the possibility of introducing a prevention campaign in the game in connection with official institutional programs. Some also considered links to existing resources, with access to information summaries.

##### Challenge as a Motivation to Participate

One of the participants proposed a call for poster production in the form of a challenge to encourage the participants’ motivation and production:

...a poster competition within the game, with the results displayed in room galleries of the game.

S8

Another participant reminded us that there are already challenges in the game for players and that we only need to reuse this system for the intervention:

Actually Habbo has been doing competitions for years, so that means just re-using the same idea, and suggesting that players make a poster about smoking.

S7

In addition, one of the participants mentioned competitions with a prize to win:

Later we could set up animation games with a monthly ranking. For example, the top 5 players could be given a badge.

S14

##### An Entertaining Intervention That May Be Considered Not Serious Enough

When we asked whether an in-game prevention message is a good idea, we were told the following:

I am not sure, Philou [the facilitator], maybe they won’t take it very seriously.

S3

A participant (S1) warned that playful or entertaining elements may not lead players to consider the prevention message integrated into the action:

I think [the over-entertaining aspect of the prevention game] may be completely counter-productive and undermine the message it is supposed to convey.

S1

##### The Involvement of Multiple Stakeholders in the Development and Implementation of Interventions

When asked about the technical development of the action, some participants mentioned the help of the community manager, present and known to all. She is the one who grants permissions and accompanies the creation of new elements of the game. She would have the task of explaining the course of a multicomponent action (S12):

So organize animators let’s say once a week in addition to a discussion, with that a monthly ranking, with ‘the question box’ for the follow-up...the news of the community manager to explain the whole system.

S12

More generally, the participants suggested involving several actors in the game for intervention: the community manager, the health professionals, and the player ambassadors (S1, S12, and S14):

I think one of you two [facilitators] should be involved, with a Habbo representative, a player, and with an event on the welcome page. That makes it a real thing that’s being organised.

S14

As a result, the interviewed participants showed a willingness to take action with their fellow players.

#### Involve Peers in the Development of the Intervention in the Game

The thematic analysis highlighted the community dimensions within the game that can be levers of attraction to preventive action. These elements were organized into 5 subthemes.

##### In Favor of Codeveloping the Action

Most participants expressed a willingness to contribute to the development of preventive action within the game. Their role can be that of being a peer advocate or peer educator:

If it can have an impact on other people and can help them, I would be very happy to take part…I would hope to be able to take part or be a facilitator, to interact with the players and in fact help them with topics.

S2

##### Remain Anonymous for the Fear of Not Being Taken Seriously

If they were to be involved as peer educators in delivering actions, 3 participants (S8, S12, and S14) mentioned the need to be anonymous with a different pseudonym or avatar. Not being recognizable as a classical player would make other players listen to them more. One of the participants explained the need for a professional presence to be taken seriously:

In fact the worry is that if there are no professionals with us the players won’t take us so seriously, because we know each other.

S12

Indeed, some are well known by their game peers, and it would be difficult for them to be taken seriously:

On the other hand if I appeared as a clone that would be fine, because I wouldn’t be recognized. So long as there is anonymity I have no problem.

S14

##### Feeling of Insufficient Knowledge for Delivering Prevention Themselves

Beyond the players’ self-projection as prevention actors, one of the participants emphasized his feeling of not being able to intervene on all health issues:

I don’t feel I am capable of making a judgment about everything.

S13

He also pointed to his lack of experience in talking about certain topics:

Personally I don’t think I am the best person [to do smoking prevention] since I have never smoked.

S13

##### Experience as a Skill for Prevention

Participants mentioned that experience then intervenes as a skill that can be mobilized by the players or professional facilitators (S1, S12, and S15):

Yes, when it’s doing adapted prevention, which can also bring out your personal experience of the subject.

S1

...with people who have been affected by the subjects being discussed perhaps.

S15

I am not a good example although being a smoker myself I know all about the downsides.

S12

If mobilized for delivering prevention messages, a participant reported that his point of view would be more intelligible and impactful:

Our personal experience will make it more impactful...I would have more to talk about and have more impact in what I say.

S2

##### Motivation and Social Support Among Peers as a Lever for Action

For a participant (S6), the involvement of peers could take place through a social support system between gaming peers through sponsorship, similar to existing support strategies outside web-based games:

We could do it like our counterparts for alcohol, except that we are on Habbo...Yes, the units could be a series of badges and they can be used as tokens, like for alcoholism.

S6

This peer-to-peer social support can lead to web-based group challenges, such as the community challenge mentioned by one of the participants:

One could also think about a community challenge with ranking results.

S8

However, another participant (S11) highlighted the difficulty of motivating and mobilizing other players through digital means, especially for prevention:

Well, yes I would be happy to help someone stop smoking, but it’s tricky sitting with a PC, isn’t it?

S11

#### Mobilize and Control Gamer Participation

Beyond the involvement of web-based gaming peers as peer educators, the thematic analysis highlighted the ways in which players (the target audience) are engaged in prevention. In this regard, 4 subthemes were highlighted.

##### Limiting the Number of Participants to Ensure the Success of the Action

The participants expressed that it is more pleasant to play the game in small groups:

Yes, it’s more pleasant in a small committee.

S12

Several participants emphasized the need to think about a prevention action that would mobilize small groups in the game (S2, S4, S5, and S12), especially to be able to debate properly (S2). One of the participants (S5) suggested limiting the number of participants to 10, always with 2 facilitators. It was also pointed out that several sessions are necessary (S2):

With a big group (100) it’s likely to be more difficult because there will always be people to disturb others who really want to pay attention.

S2

I loved the once-weekly format.

S14

##### Trolls and Essential Moderation of Exchanges

Many players also talked about disrupters within the game (trolls). One of the participants said that there will always be “those who distract people who want to listen” (S2). A participant indicated that moderation is mandatory for the proper conduct of the action:

It’s obligatory, Philou, you were in luck here, but in the bus there are usually trolls.

S1

This participant considered ambassadors (players with good behavior in the game) as people who could support this moderation:

with one or two ambassadors to moderate the players who come to provoke everyone in your INSERM sessions.

S1

##### In-Game Rewards as Incentives

The participants discussed how to reach players and enroll them in prevention action. Some participants (S1, S9, and S10) indicated the importance of rewards for engaging players:

To be very frank with you, if there aren’t any rewards to be had, and it’s just for prevention...you won’t get many followers.

S9

The same participants pointed out that in the game, “badges” are collectibles and that many players want to acquire them:

Those who win get a badge, players really like badges.

S6

Then I also think there has to be a badge for each session depending on the theme (people will be more receptive).

S1

However, 2 participants (S3 and S11) suggested that players who come only for the incentives (badges) would be less influenced by the prevention action:

Many of them will come just for the badge.

S3

It’s true that if there are incentives to win there will be more players, but we don’t want them to come just for the badges.

S11

One of the participant (S12) expressed that not implementing these incentives would be a way to select the most motivated individuals:

Those who are prepared to attend knowing there is nothing to win are sure to be the most sincerely motivated.

S12

##### Promote Action Outside the Web-Based Game

A participant indicated the importance of taking action beyond the game, especially to reach more people:

To be honest, Habbo is not the way to mobilize the most people, you need to aim higher.

S7

Another way to get players to participate in action would be to promote the action inside and outside the game among the Habbo community (on Twitter [Twitter, Inc] and fan websites). One of the participants suggested putting billboards about the prevention action in the game (S2). Another participant (S11) suggested that the community manager could advertise on the game’s home page and through alerts.

Yet another participant suggested recording the actions to replay them and reach more people:

in a public space in the game, with the Habbo viewing experience. Film it and replay it on fan sites [for fans outside the Habbo game itself].

S1

Another participant (S6) mentioned that the social networking site Twitter and game-related accounts can be used to advertise the action:

You can do ads on Twitter, on the Habbo news site, and in the bus you can work on themes around addictions like cigarettes.

S6

### Recommendations’ Elaboration and Application

Following the analysis of young people’s proposals and professionals’ experiences, we established 16 domains of 27 recommendations for health intervention in existing web-based community games (Multimedia Appendix 2). Each illustration describes the application of each recommendation during the development of new actions introduced in the game. Thus, Table S1 in Multimedia Appendix 2 integrates the fields, recommendations, applications of the recommendations in new interventions, and descriptions of specific new interventions. Multimedia Appendix 3 describes the interventions developed in response to these recommendations.

## Discussion

### Principal Findings

Our study highlights Habbo players’ proposals for developing interventions within the web-based game. These proposals emphasized the importance of serious interventions that need to involve and moderate a small group of people in a playful way. Using a reflexive approach, we also proposed recommendations for application in different interventions in general web-based community games. These recommendations highlight numerous suggestions for preparing and implementing an intervention, correctly incorporating it into web-based games, and adopting the codes of the web-based community game culture.

### Meaning of the Findings

Our study showed that it is possible to work in fields that are not commonly explored and exploited by intervention research, with important recommendations for making good use of them. This study shows that it is possible to integrate the intervention into games that the target population already plays instead of creating a new game for a health intervention. We knew that it was possible to intervene in the game (following the Fil Santé Jeunes experience). Nevertheless, this study wanted to delve further by collecting concrete proposals from the players. Recommendations based on the Fil Santé Jeunes experience completed these proposals. This made it possible to bring together the contributions of various stakeholders (target population and professionals) via a participatory research approach [24,25].

The players indicated the importance of addressing a variety of health topics, that is, being responsive and diverse in health promotion responses. The study participants highlighted the importance of addressing many of the associated themes in health promotion action in a web-based game. More generally, a large number of web-based games are used for individual health, mainly for cognitive training or indirect health education [26], physical activity [27], managing chronic disease [28], preventing bullying [29] or preventing tobacco use [30]. Health actors have developed a very large number of games, and these games are generally novel interventions delivered to individuals, with different degrees of acceptability, attractiveness, and feasibility.

The participants highlighted the need for different types of involvement (the involvement of peers and facilitators) and ways to conceptualize and implement interventions for health. Web-based games can play a major role in the adoption of positive health behaviors, particularly because they promote players’ self-esteem, provide support and information, and facilitate interaction and communication [28] with a sense of community. The world of games can be a real support. For example, avatars are important among young people as an extension of the self or as a way to further experiment with social relationships safely. A previous study has shown that among trans or gender-diverse youth, the use of an avatar facilitates the expression and consolidation of gender identity, with powerful mental health benefits [31].

We proposed several recommendations on how to act in existing games, including the consideration of participants at several levels: personalization, solicitation, animation, and moderation. Furthermore, in the field of mental health, Fleming et al [32] made the following recommendations for using web-based games for interventions [32]: a user-centered approach requiring exploration of group preferences, engaging and effective interventions with a description of game dynamics, cross-sectoral and international collaborations to seek out and identify the skills required for game development, and rapid testing and implementation to keep interventions engaging.

From our perspective, we highlighted new elements to intervene in already existing web-based games. Nevertheless, the literature still has a few examples of interventions integrated into existing games that are popular with the target audiences. Few studies have focused on the added value of using games rather than creating them, whether in response to health issues or economic issues related to development [33]. One of the studies, however, highlighted the use of existing commercial video games as therapies, studying these games as potential health interventions [34]. Colder Carras et al [34] noted that there is a lack of research on the use of commercial games for health and highlighted the need to design standardized protocols, study best practices to identify which games work on which health conditions, and model the complex and dense data of games. In the future, it will be possible to understand the effectiveness of actions integrated into web-based games for health promotion by considering the influence of action implementation and processes in web-based community games.

### Implications for Decision Makers and Practitioners

Decision makers and initiators of health promotion interventions must consider several approaches to intervene through web-based games beyond the community aspects described in this study. It is necessary to consider that it is possible to intervene by 3 means: fitting the intervention into an existing game, creating a game specifically to intervene, and reusing a game without modification.

For interventions in existing games, it is necessary to consider what is practically possible (the potential of action). It remains necessary to understand the game’s functioning to provide adapted interventions (functioning and adaptability). Some games do not allow for this kind of intervention because there is too strong a difference between the game objectives or gaming companies’ culture and health objectives (real feasibility). If an intervention is possible, it is necessary to acculturate the intervention to the codes of the game without modifying the game. This requires the training and adaptation of external stakeholders (acculturation). Moreover, intervening in a game is only possible if there is a good link between the developers and stakeholders. It is important to establish a strong and trusting relationship with developers, particularly to integrate interventions in games (relationship). This relationship should also be considered from a research perspective, as it involves considering the data to be analyzed beyond the intervention to be implemented. On the one hand, the researchers must present to developers the real issues of research and the importance of collecting data that can be analyzed according to sometimes rigorous and complex methodologies (such as randomized controlled trials). On the other hand, the developers must explain how the game works (in a way that does not have health promotion as its initial goal) to anticipate both the best potential for action and the way in which the research can be carried out (eg, acceptability and accessibility of participants’ data).

To develop a game for a health promotion intervention (similar to Hong et al [35]), it is essential to consider the levels of involvement needed. It is important to consider the need for technical skills to develop and maintain the game (technical skills). It is also important to consider the costs inherent to development and maintenance (costs). Moreover, it is essential to understand the uses of, expectations for, and attractions of the games (understanding), particularly the benefit of creating a new game (innovation). Developing a web-based game also takes time, with the risk of obsolescence of the game in the face of rapidly changing preferences (temporality and obsolescence). Creating a web-based game for health also raises the issue of reaching target audiences, who are already engaged with existing games.

**Figure 3:**
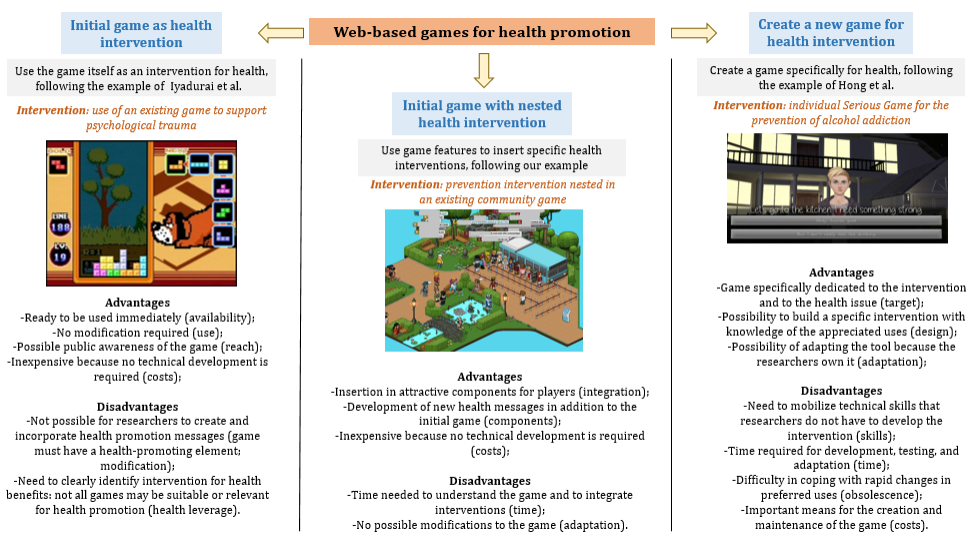
Illustration of the different possibilities to intervene through web-based games (Hong et al [35] and Iyadurai et al [36]).

According to the example set by some research [36], a third mode of intervention could be the replay of a web-based game as it is without inserting health content or even modifying the game’s functionality. Although this would be inexpensive, it would require a clear identification of the actual health benefits that an unmodified, commercial web-based game could provide. The integration of these games with offline health practices or services is also generally required. These 3 modes that we discussed are illustrated in Figure 3.

In general, it is important for stakeholders to know which strategy is the most relevant to the target population, considering its accessibility and expectations (realism). The health intervention may have objectives that allow incorporation into existing games or require the creation of new web-based spaces. In any case, it is important to consider the implementation stages, stakeholders’ roles (positions), and way to integrate populations. Incentives, elements of web-based games that can promote participation, can be used to integrate populations (incentives). In our experience, badges were attractive. The accomplishment of tasks and its valorization in game are sources of motivation for participation. For example, compensation with game’s money can be discussed.

### Strengths, Limitations, and Future Directions

Our study is one of the few to investigate the potential of existing web-based community games for health promotion. It provides concrete methodological recommendations for the conceptualization and implementation of health actions in existing games. However, this study has limitations. We consulted the participants who were involved in our intervention, as the questions asked in this study occurred in the fourth session of the intervention, inducing a selection bias and not reaching the most distant players. In fact, we do not have information about the preferences of those who are more difficult to reach in the game. However, we collected the opinions of the most motivated players; they discussed the attractive elements of intervention for all players and how to develop and implement interventions for the most distant players. We developed our recommendations on 1 kind of web-based game (community based, with the possibility of development of elements in the game by the players).

We developed recommendations according to a structured methodology based on the experience and analysis of the project researchers. This methodology and the application of these recommendations in the development of new interventions demonstrated the internal validity of the recommendations. Recommendations must nevertheless be tested and validated in other games of this type, by other facilitators, with other intervention components, and for other health issues. Furthermore, the various functionalities offered by different web-based games require tempering the application of certain recommendations. Indeed, in our case, the game is mainly a place of web-based social interactions with gamification elements. Other web-based games might have features that are less community based. Therefore, our article focused on web-based community games with a socialization purpose. In addition, some of the recommendations may also be applicable outside existing games—not only to the development of new community health games but also to interventions using facilitation, communication, and participation techniques for health promotion.

Future research must continue to understand the processes involved in this type of intervention, depending on the audience and health issues being addressed. Indeed, interventions in web-based games can vary by topic (eg, differences in appeal between tobacco and mental health actions and differences in behavior change theories) and by participating audiences. It is still important to understand which population is using different popular web-based games to better understand whom we are reaching and what efforts need to be made. Knowing which population is attracted to health promotion activities remains important. Integrating target audiences (young people) into research processes remains essential in a community-based participatory research approach [24,25]. This will enable a better understanding of how to adapt and develop acceptable and attractive health promotion interventions in web-based games. Finally, research must involve evaluations based on complex intervention research recommendations to measure the effect of health promotion interventions in games on health determinants targeted by researchers and actors (information, attitudes, skills, and behaviors), taking into account context effects. To do this, the methodologies must be designed in an interdisciplinary manner and be based on existing recommendations [12,37].

### Conclusions

Health interventions integrated in existing web-based community games represent a potential for promoting individual health and well-being. It is necessary to adapt to the changing preferences and uses of target populations by thinking of innovative means for intervention research (levers, brakes, and costs). This also requires a permanent acculturation of the interveners and researchers to correctly intervene and analyze the games as a place and potential for health promotion among the target audiences. Involving these audiences is of considerable help in designing and implementing innovative health promotion interventions.

## Appendix

Multimedia Appendix 1 Description of the theoretical framework of the Scott intervention.

Multimedia Appendix 2 Key recommendation elaboration and application for health promotion intervention in a web-based community game.

Multimedia Appendix 3 Description of interventions created based on the recommendations.
