# Supplementary material for: Health Promotion in Popular Web-Based Community Games Among Young People: Proposals, Recommendations, and Applications
Source: JMIR Serious Games. 2023 Jun 9;11:e39465. doi: 10.2196/39465 (PMC10337365; doi:10.2196/39465)
Supplement: Multimedia Appendix 1 [file games_v11i1e39465_app1.docx]

**Multimedia Appendix 1**

The theoretical framework of the Scott intervention integrates both behavioral theories related to health behaviors in youth, but also social theories related to online interaction, participation and collective motivation. The theoretical framework takes into account theories of how online communities function (social roles established, interaction patterns, interest in features and content).

1. The theory of planned behavior [15] incorporates both social influences and personal factors as predictors, specifying a limited number of psychological variables that can influence behavior, namely 1) intention; 2) attitude; 3) subjective norm (SN); and 4) perceived behavioral control (PBC). Subjective norms are conceptualized as the pressure people perceive from others to perform a behavior. People's positive or negative evaluations of their behavior are conceptualized as alternative predictors of intention (attitudes). Perceived behavioral control represents an assessment of how easy or difficult it is to perform the behavior, and it is thought to reflect the obstacles one has encountered in past behavioral performance. Finally, attitudes, subjective norms, and perceived behavioral control are proposed to influence behavior through their influence on intentions, which "summarize a person's motivation to act in a particular way and indicate how much the person is willing to try and how much time and effort he or she is willing to devote to performing a behavior." For the Scott intervention, the choice of behavior change theory was to incorporate social dynamics as a determinant of change.
2. Social learning theory [16] postulates that a health behavior (e.g., smoking) is a behavior learned through social interaction and reinforcement [19]. Individuals learn from each other through observation, imitation and modeling. This theory has four stages, which are attention, retention, reproduction and motivation. A youth's participation in substance use models likely has three effects, beginning with observation and imitation of substance-specific behaviors, continuing with social reinforcement (i.e., encouragement and support) of experimentation with substance use, and culminating in the adolescent's expectation of positive social and physiological consequences from that experimentation [19]. Expected consequences may be largely social in nature during experimentation (in the form of peer acceptance or rejection). This theory concludes by stating that an adolescent who expects substances to produce more personal benefits than consequences will be at risk for experimenting with substance use. In the Scott intervention, group sessions will aim to develop a shared commitment and social reinforcement to promote supportive behaviors. In contrast, the focus here is on creating social support to encourage positive behaviors. Attention is paid to how the social group is formed within the intervention and how social norms are constructed.
3. Among the many existing educational approaches, peer education [17] is defined as "an educational approach that uses peers (people of similar age, social background, function, education or experience, in this case "peer gamers") to provide information and to emphasize types of behaviors and values" [20]. Peer education is a process by which well-trained and motivated individuals undertake informal or organized educational activities with their peers to develop knowledge, attitudes, beliefs and skills that enable them to be responsible and protect their own health. Youth would be a very receptive population for this type of education, especially in light of existing social dynamics, beyond the simple health promotion approach. Young people could then receive support from their peers, but could also be actors in a certain reciprocity.

Link between peer education and social learning theory [18]: In terms of claims for peer education, social learning theory seems to be relevant in terms of credibility, empowerment, role modeling, and reinforcement. Peer leaders could have credibility with others to be influential. To act as role models, according to the principles of the theory, peers would need to be able to observe peer models practicing a health behavior. Peers would then need opportunities to practice it themselves and would need positive reinforcement. For the Scott intervention, the goal is to strengthen the social group of peers, to get them to disseminate positive social norms about positive health behaviors.

1. Small groups and behavior change [14]: We distinguish here between the dynamics of online communities according to the size of the groups within the community. In the Habbo community, our intervention is designed for small groups of 10 participants (repeated sessions to have 30 participants in the intervention group). Based on a review of the literature, Borek and Abraham developed a conceptual model of mechanisms of change in small groups. Five categories of interacting processes and concepts were identified and defined: (1) group development processes, (2) dynamic group processes, (3) social change processes, (4) personal change processes, and (5) group design and operating parameters. Each of these categories encompasses a variety of theoretical mechanisms explaining individual change in small groups.
