# Supplementary material for: Health Promotion in Popular Web-Based Community Games Among Young People: Proposals, Recommendations, and Applications
Source: JMIR Serious Games. 2023 Jun 9;11:e39465. doi: 10.2196/39465 (PMC10337365; doi:10.2196/39465)
Supplement: Multimedia Appendix 2 [file games_v11i1e39465_app2.docx]

# **Multimedia Appendix 2.** Key recommendation elaboration and application for health promotion intervention in a web-based community game.

|  | Domain and the Scott intervention experience | | Recommendations | Applications for new interventions |
| --- | --- | --- | --- | --- |
| **Preparation** | | | |  |
|  |  | 1. The project team (researchers) built the content of the action, along with members with several skills: tobacco prevention professionals, psychologists who have experience in the game, prevention facilitators, sociologists, and public health researchers. | 1. Build a complementary and multidisciplinary team to understand the public health problem and the target audience and define the objectives of the health promotion intervention. | 1. The facilitators had to define the health issue and draft the health promotion message to share them with the players. They had to mobilize validated scientific sources and contact specialist professionals. For example, to deal with sedentary lifestyles, they developed a challenge in the conversation to practice sports after a motivation session in the intervention. This challenge was based on medicine courses and scientific literature. |
|  |  | 2. The members of the project had to identify, prepare, and study the functionalities and specificities of the selected web-based game to build an adequate and feasible action. | 2. Learn about the culture and universe of the game to conceptualize the intervention adapted to the technicality of web-based games. | 2. The new facilitators received information on the purpose (community aspect) and the functioning of the game. They were able to ask questions about the interests of the players, especially to intervene in the best possible way. |
| **Collaboration** | | | |  |
|  |  | 3. The team identified the web-based game Habbo, which is aimed at young people. We approached the company in charge of developing the game to propose and contractually establish a collaboration to intervene in the game. | 3. Define from the outset the relationship with the commercial partner (company developing the game) at the level of collaboration (agreement of the partner, time allowed, and availability) and its conditions (costs, terms of action, data to be collected, and level of involvement). | 3. Positive past experience enabled coming up with new interventions and confirming agreements with the owners of the game to continue new health promotion interventions. |
|  |  | 4. We worked directly with the community manager to develop the functionalities of the action (bus, room, and operation) and modalities of the study (questionnaire display and randomization). Before implementation, a test phase was conducted with the community manager and ambassador player to verify the operation of the intervention. | 4. Work from the beginning to end with the game community managers (or other game stakeholders) to correctly design, test, and implement the action and research study. | 4. The new facilitators developed a new relationship with the community manager (with former facilitators), and they developed new interventions with peer ambassadors. The knowledge of the former facilitators allowed the peer players to make the connection and continue the relationship with confidence. |
|  |  | 5. Players helped the facilitators create badges for the intervention based on the chosen health theme of the moment. | 5. Allow players to contribute to the preparation of the intervention by permitting them to create the objects and places of the intervention themselves. | 5. The players built new attractive places for the intervention with the facilitators: gym, restaurant, room, and bar. This made it possible to adapt the interventions to the expectations of the players and brought elements of attractiveness. |
| **Formation** | | | |  |
|  |  | 6. The facilitators registered for the web-based game, developed their avatar, and learned about the game’s specific controls to anticipate the inherent workings and interactions. | 6. Learn about the web-based community game (environment, maneuvers, codes, and techniques) to master it at the time of the action design and implementation. | 6. The new facilitators repeated the learning phase, with a transmission of experience by the first facilitators and the accompaniment of the peer ambassadors and the community manager. |
| **Identification** | | | |  |
|  |  | 7. Stakeholders developed their avatar with a white coat and a pseudonym containing the word “health” (eg, eno-santé) to be seen by other players as professionals in the field. | 7. Develop a web-based persona (avatar) identified as a professional actor in the field of prevention, education, or health promotion to be recognizable and acknowledged in the game. | 7. The facilitators used the term “health” in their pseudonyms, which brought a serious dimension and provided a reminder of the previous positive intervention (confidence in the first facilitators). Facilitators explained their medical curriculum. They presented themselves as medical students with an expertise in health issues. |
| **Content** | | | |  |
|  |  | 8. The project actors proposed 4 sessions around smoking prevention with the following topics: benefits and drawbacks of smoking, expectations of young people regarding prevention within the game, resisting the incitements of the entourage, and taking on challenges and working for others. | 8. Prepare in advance the contents and the course of actions to carry out in the web-based game to bring a structure of action in a very dynamic web-based game. | 8. The facilitators prepared their interventions in groups. They discussed the health promotion messages to be integrated into interventions: definition of the problem, information to provide, and modalities of dissemination (quiz and challenge). They discussed, adapted, and validated these contents with facilitators before implementation. |
| **Research** | | | |  |
|  |  | 9. The researchers prepared in advance the questionnaires they wanted to administer to the participants, and they planned how to administer them with the community manager. They also discussed how to set up individual randomization to create an intervention group and a control group. | 9. Prepare questionnaires and other research tools and conditions in advance and plan how to position them in the game. | 9. No research documents or tools were planned for these interventions. |
| **Advertisement** | | | |  |
|  |  | 10. A week before the first intervention, the community manager published a banner developed with the researchers. A schedule and timetable was announced in this advertisement. | 10. Prepare an announcement system within the game to create a phenomenon of expectation around the intervention and allow users to plan their arrival in the intervention. | 10. The same announcement system was provided, indicating that interventions of the same institution (Inserm) would return in Habbo for new interventions. The advantage was that the players knew the Scott intervention and had a good feeling about it, which made them want to participate again. |
| **Recruitment** | | | |  |
|  |  | 11. A room (a garden to be precise) was built as a waiting space, where players could wait and interact with the facilitators before the action took place in the dedicated space (the bus). They could ask questions about the topic, the process, and the schedule. It was in this space that participants were recruited. | 11. Interact with players before starting the intervention to raise interest and emphasize the importance of their participation. | 11. The facilitators asked for the development of a common place (garden) to access the different interventions (different buses, giving access to the places of intervention, which can be a restaurant, bar, or sports hall). The garden allowed for welcoming the future participants and explaining the interventions to them. The facilitators began to interact with the players before the intervention to invite them to participate. The former facilitators (supervisors) were also present in the garden to meet former participants and be recognized in this place, ensuring more confidence. |
|  |  | 12. The facilitators let the participants come to the venue on their own in the intervention. | 12. Determine a passive or an active recruitment method: let future participants come on their own to the intervention or bring them  into the game to directly invite them. | 12. The facilitators first let the players arrive at the intervention site on their own. If the participants who came on their own were enough, the intervention could begin. If there were not enough participants to deliver interventions, the facilitators explored the game to actively recruit players for the intervention. |
| **Implementation** | | | |  |
|  |  | 13. A bus specially designed for the intervention was developed with the community manager. This bus was recognizable and included fun and game-specific elements (chairs and drinks). | 13. Provide a dedicated, a recognizable, and an attractive place for intervention implementation within the game. | 13. The new facilitators integrated elements of the game used for the Scott intervention (bus) and also adapted their new interventions using new varied and attractive places (eg, gym and restaurant). This has allowed them to maintain familiarity and originality. |
| **Temporality** | | | |  |
|  |  | 14. The dates and duration of the interventions were announced in the advertisement on the home page and recalled in the intervention. This allowed the participants to be prepared for the beginning and the end of the interventions. | 14. Plan and announce the duration of the intervention within the game to prepare the participants to get involved in the game at a given time. | 14. The system for announcing new interventions included a schedule according to the different health themes addressed (sexual health, addictions, nutrition, and physical activity). |
|  |  | 15. The duration of the interventions was limited to a maximum of 30 minutes. | 15. Keep the time short to sustain participants’ interest and not to distract them from their initial goal of playing the game outside the intervention | 15. The facilitators proposed 5 days with 5 interventions per day, with each intervention lasting for 40 minutes (20-minute break). They announced the longer duration. |
|  |  | 16. Thirty-minute sessions were scheduled at different times (11 AM, 3 PM, and 5 PM) to accommodate the schedules of different players. | 16. Propose several different times to adapt to the players’ schedules. | 16. The times of new interventions varied between morning, lunchtime, afternoon, and evening. |
| **Rehearsal** | | | |  |
|  |  | 17. Four sessions were planned over a month for the same group, with 1 session per week, allowing the establishment of a periodicity familiar to the players (3 groups were formed). | 17. Provide a repetition of the action within the web-based game: multiple action sessions for 1 group (also ensure the repetition of action sessions for other groups). | 17. Facilitators were present over 5 days per week. Some participants were able to attend 1 session per day, thus receiving a repeated and diversified intervention. Two weeks of intervention were scheduled, with a 1-week break in between. |
| **Adaptation and personalization** | | | |  |
|  |  | 18. The intervention was adapted in light of the topics discussed by the participants in the action to allow for a personalized prevention response. | 18. Adapt one’s health promotion intervention during and after the intervention to maximize the relevance of the intervention within the web-based game. | 18. All the facilitators had to adapt their intervention after the initial stages of the intervention: find new topics and new ways to communicate. For example, the facilitators had to create new content on sports for the physical activity theme because the players were already familiar with the initial content (owing to coming back regularly). |
| **Animation: between familiarity and seriousness** | | | |  |
|  |  | 19. The facilitators used terms specific to the features of the Habbo game (eg, mute, kick off, and terms corresponding to game features). | 19. Adopt the language expressions and codes of web-based game players “to speak” the same language spoken by them. | 19. The new facilitators also adopted the same language expressions. |
|  |  | 20. Facilitators could use youth language and familiarity without forgetting their role as facilitators and moderators (regular reminders). | 20. Maintain the posture of a health professional to ensure a correct framework with an attitude between seriousness (ensuring confidence) and familiarity (ensuring playful aspect). | 20. The new facilitators could be overwhelmed by the familiarity of the intervention and, therefore, had to reimpose a professional posture. |
| **Moderation** | | | |  |
|  |  | 21. The facilitators planned a facilitation with the following elements: (1) a reminder of the rules of respect, (2) questions for interaction, and (3) a thank you and an end dance (game feature) at the end of each session. | 21. At the beginning of the intervention, announce the rules and the risk of exclusion from the intervention in case of inappropriate behavior. | 21. The facilitators reminded the players of the rules in the same way, insisting more on the risk of exclusion if they misbehaved. |
|  |  | 22. Interveners were to exclude disruptive elements, such as insults, inappropriate language, or bullying. Disruptive players were warned once and excluded if there was no noticeable change in their behavior. | 22. Ensure a moderation system for game participants’ interactions (with target audiences among themselves and with speaker), with the possibility of warning or excluding trolls from the intervention. | 22. The animators excluded players with bad behavior. The exclusion threshold was different for each group, requiring a framework to be set beforehand for each group. Moderation was sometimes not done enough before exclusion: some players were provoking but asked to be accompanied in the intervention and not excluded. |
|  |  | 23. Player ambassadors could access exclusion features to help facilitators and moderate disruptive elements. | 23. Mobilize players present in the intervention for peer moderation of disruptive elements. | 23. The ambassador players remained present and were more active because more people were present in the new interventions. |
| **Targeting participants** | | | |  |
|  |  | 24. In the general room, the facilitators addressed the participants using their game’s alias before speaking. The same was done in the prevention action in the dedicated bus (eg, Eno-santé: “xx-xxx ah yes? What did you want to say?”; Philou-santé: "xxxxx, what do you think of what the others say?) | 24. In-game general navigation and in-game intervention: calling out or mentioning the participants you are addressing strengthens interaction, encourages participant expression, and builds trust. | 24. The facilitators reused this targeting system. |
| **Building community with action** | | | |  |
|  |  | 25. We planned a special event (party) for the completion of the 4 intervention sessions, where all participants (control and intervention groups) were invited to dress up (Halloween time) and interact with the invited interventionists. | 25. Build community around the action in the game so that players have a sense of familiarity with health promotion interventions. | 25. The facilitators had prepared a challenge in the interventions. A party-like event was also organized at the end of the interventions, with an announcement of the winners of the contest. |
| **Reputation** | | | |  |
|  |  | 26. At the end of the sessions, the facilitators told the participants to “feel free to publicize us to other Habbos.” Former players would come back into the room to explain to other players that they enjoyed the action. A Habbo Twitter account was maintained by “ambassador” players. These players took pictures of the end-of-session party and posted them on Twitter. | 26. Capture the reactions of the participants so that they can positively publicize the action and its future actions, both within and outside the game. | 26. Players repromoted past interventions by posting them on external and game-associated social media. |
| **Incentives** | | | |  |
|  |  | 27. The study participants had a badge before and after participation. These badges have a strong incentive value. | 27. Understand what incentives can be deployed in the game and incorporate them as a motivator. | 27. At the end of the challenges in interventions, the community manager provided additional badges for the winners. |
