# Supplementary material for: Health Promotion in Popular Web-Based Community Games Among Young People: Proposals, Recommendations, and Applications
Source: JMIR Serious Games. 2023 Jun 9;11:e39465. doi: 10.2196/39465 (PMC10337365; doi:10.2196/39465)
Supplement: Multimedia Appendix 3 [file games_v11i1e39465_app3.docx]

# Multimedia Appendix 3 - Description of interventions created from the recommendations

**Facilitators**: 8 trios (24 facilitators) have been trained to intervene in the Habbo game. These facilitators were in their 3rd year of medical school.

**Preparation**: each facilitator received training on the functionalities of the game. Each trio had to propose a health intervention based on the game features (e.g. quiz, discussion group). They also had to define prevention and health promotion messages on themes of interest (e.g. information on risky consumption, information on sexual and gender identities). They had to anticipate how these messages would be disseminated and how the moderation of the exchanges would follow (e.g., if violence was expressed, solution to provide help numbers).

**Temporality**: 4 trinomials were planned per week over 2 weeks (8 trinomials in total). These trinomials intervened for 5 hours in a row in the game, with intervention times varying from 30 to 50 minutes. The 4 trinomials of the same week had different intervention times, making the health interventions in the game accessible from 11am to 8pm.

**Places developed**: for both weeks, a general garden was created to accommodate 4 buses dedicated to the 4 interventions of the week (see illustration). A queue was provided for each bus.


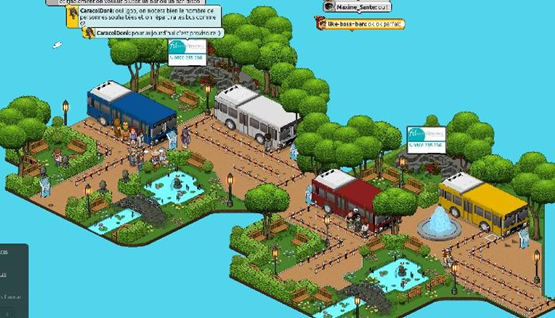


**Illustration 1 – general garden created for access to interventions**

These buses gave access to different rooms according to the trinomials of animators and their developed interventions (cf. illustration). These rooms differed in their activities and functionalities but also in their themes (see table).

| **Addictions (night club)** | **Sexual health (love room)** |
| --- | --- |
| 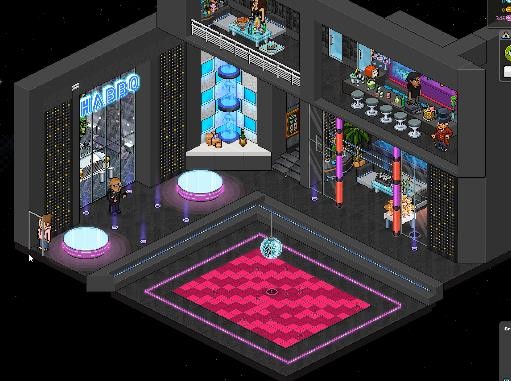  Action : Treasure hunt and quiz | 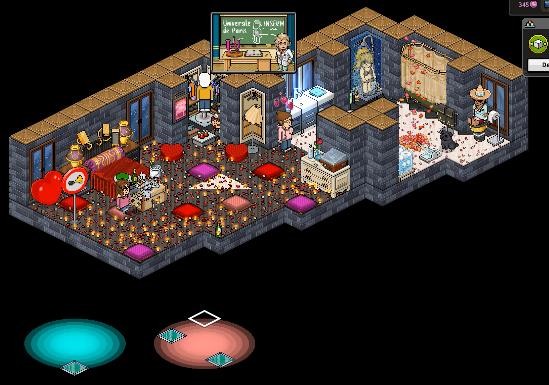  Action : Chat and quiz |
| **Alimentation (restaurant)** | **Physical activity (gym room)** |
| 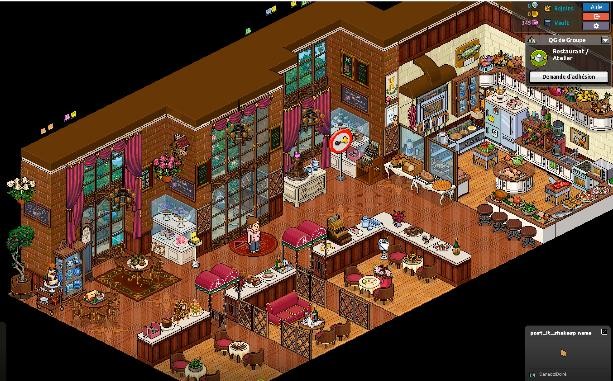  Action : Chat and quiz | 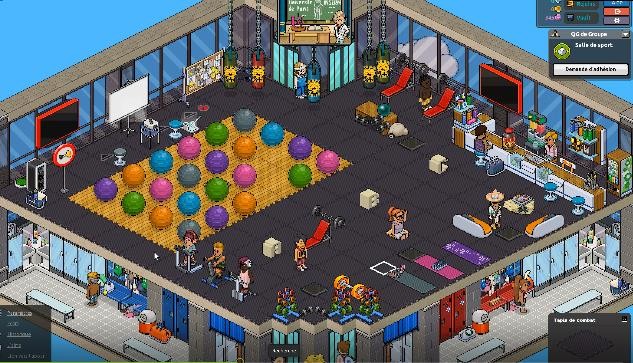  Action : Chat and quiz |

**Illustration 2 – Examples of new places developed**

**Table 1 – Health subjects integrated in interventions**

| **Phsyical activity** | **Addiction** | **Alimentation** | **Santé sexuelle** |
| --- | --- | --- | --- |
| **Facilitators 1 :** Sedentary lifestyle, physical activity and sport, cardiovascular disease, vascular disease, sleep | **Facilitators 2 :** Alcohol (main theme). Drugs, tobacco and screen addiction | **Facilitators 3 :** Balanced and accessible meals at low prices | **Facilitators 4 :** Sexual transmitted infections, identity and gender, sexting and the couple, consent |
| **Facilitators 5 :** Sedentary lifestyle, physical activity and sport, cardiovascular disease | **Facilitators 6 :** Alcohol, drugs, tobacco and screen addiction | **Facilitators 7 :** Balanced meals, macronutrients/micronutrients, nutritional contributions of foods | **Facilitators 8 :** Pornography, sexting, pleasure and desire |
